# P3H4 Enhances the Proliferation, Invasion, and Glycolysis of Hepatocellular Carcinoma Cells

**DOI:** 10.1155/ijog/2532296

**Published:** 2025-11-09

**Authors:** Lili Yan, Yandan Fan, Ji Lv, Meimei Xu, Hongyu Jia, Shanshan Li, Zhihui Tan, Chunyan Lin

**Affiliations:** ^1^ Department of Gastroenterology, First Hospital of Qinhuangdao, Qinhuangdao, China, qhdsdyyy.com; ^2^ Department of Medical and Radiation Oncology, Affiliated Sanming First Hospital of Fujian Medical University, Sanming, China, fjmu.edu.cn; ^3^ Department of Breast Surgery, First Hospital of Qinhuangdao, Qinhuangdao, China, qhdsdyyy.com

**Keywords:** glycolysis, hepatocellular carcinoma cells, invasion, P3H4, proliferation

## Abstract

**Objective:**

The study is aimed at investigating the functions of P3H4 in liver cancer.

**Materials and Methods:**

The TCGA and CPTAC liver cancer databases were utilized to analyze the expression levels of P3H4 and its associated survival rates between tumor and normal tissues. A cohort of 60 HCC patients was selected based on criteria for P3H4 expression and survival analysis. qRT‐PCR and western blotting techniques were employed to assess P3H4 expression in cell lines. The CCK8 assay was conducted to compare cell viability between normal and P3H4‐knockdown HCC cell lines. A glycolysis detection kit was used to measure glucose, lactate, and ATP levels following P3H4 knockdown. A nude mouse tumorigenicity assay was performed by injecting Huh7 cells treated with sh‐NC or sh‐P3H4; tumor volume and weight were recorded, and tumor tissues were collected for IHC analysis.

**Results:**

P3H4 expression was found to be elevated in liver tumors compared to normal tissues, as indicated by both database analyses and patient data. High expression of P3H4 correlated with poorer survival and prognosis in patients. Knocking down P3H4 significantly inhibited HCC proliferation, invasion, glycolysis, and PI3K/AKT phosphorylation in HCC cell lines. Results from the nude mouse tumorigenicity assay demonstrated that the average tumor volume, tumor weight, and the percentage of Ki‐67 positive cells were significantly reduced in the sh‐P3H4 group compared to the sh‐NC group.

**Conclusion:**

P3H4 promotes HCC proliferation, invasion, and glycolysis through the PI3K/AKT pathway, providing new insights into the diagnosis and treatment of HCC.

## 1. Introduction

Liver cancer poses a significant threat to human lives worldwide, with projections indicating that more than 1 million individuals will be affected annually by 2025 [1]. Hepatocellular carcinoma (HCC) is the most prevalent form of liver cancer, primarily caused by the Hepatitis B virus (HBV), Hepatitis C virus (HCV), chronic alcohol consumption, nonalcoholic steatohepatitis, and genetic mutations [2–5]. China has the largest population of HCC patients, largely due to high rates of HBV and HCV infections, and faces substantial challenges in extending patient survival and improving prognoses [6]. The primary treatment guideline for HCC consists of surgical intervention combined with molecularly targeted therapy; however, due to the recurrence and metastasis of HCC, patient survival and prognoses remain suboptimal [7, 8]. Recent studies have aimed to elucidate the pathogenesis of HCC, highlighting the urgent need for the discovery of new biomarkers for early diagnosis and treatment of the disease.

The prolyl 3‐hydroxylation (P3H) family a well‐conserved group of proteins involved in posttranslational modifications of collagen [9]. One member of this family, Prolyl 3‐Hydroxylase Family Member 4 (P3H4), is a nucleolar protein that was initially identified as an autoantigen associated with interstitial cystitis [10]. Research has demonstrated that P3H4 plays a crucial role in various biological processes, including tumorigenesis, cell proliferation, and immune responses [11–14]. Recent studies have indicated that P3H4 can promote the proliferation and invasion of bladder cancer, correlating with its clinicopathological features and prognosis [12, 13]. Additionally, P3H4 has been identified as a prognostic factor in lung cancer [14]. However, in renal cancer, P3H4 appears to have opposing roles. Wan et al. reported that P3H4 inhibits the development of renal cancer through the regulation of miRNAs [15], while Tian et al. found that P3H4 promotes renal cancer progression via the GDF15‐MMP9‐PD‐L1 axis [16]. These findings suggest that the functions of P3H4 in tumor progression are context‐dependent and multifaceted.

Cancer cells require glucose as a primary energy source for replication, proliferation, and metastasis. Consequently, glycolysis is recognized as a hallmark of HCC, signifying cancer proliferation, immune evasion, invasion, metastasis, angiogenesis, and drug resistance [17]. This process is primarily regulated by the AMPK/mTOR and PI3K/AKT signaling pathways. Suppressing glycolysis may represent a novel therapeutic target for HCC treatment, potentially improving patient outcomes [17]. However, the role of P3H4 in promoting glycolysis in HCC remains unclear. In this study, we conducted a series of assays to investigate the functions and mechanisms of P3H4 in HCC progression.

## 2. Materials and Methods

### 2.1. Cell Culture

Human hepatocyte cell line THLE‐3, along with the human HCC cell lines Huh7, HCCLM3, and Hep3B, were obtained from the American Type Culture Collection. The THLE‐3 cell line was maintained in Minimum Essential Medium (MEM) from Gibco supplemented with 10% fetal bovine serum. In contrast, Huh7, HCCLM3, and Hep3B were cultured in Dulbecco’s Modified Eagle Medium (DMEM) from Gibco, also with 10% fetal bovine serum. All cell lines were maintained in a humidified incubator at 37°C with 5% carbon dioxide (CO_2_) and supplemented with penicillin–streptomycin antibiotics.

### 2.2. Database Analyses

Gene expression data were obtained from the TCGA‐HCC RNA‐seq datasets and the CPTAC datasets (http://ualcan.path.uab.edu/cgi-bin/CPTAC-Result.pl?genenam=P3H4%26ctype=Liver), which include 529 samples (369 tumor and 160 normal samples) and 330 samples (165 tumor and 165 normal samples), respectively. The RNA‐seq data were analyzed using Gene Expression Profiling Interactive Analysis (GEPIA) to compare gene expression between tumor and normal samples. Additionally, survival analysis was conducted using the K–M plotter analysis, with overall survival (OS) considered the endpoint for the patients. A total of 60 HCC patients were recruited in accordance with the requirements set by the Qinhuangdao First Hospital Ethics Committee. Written informed consent was obtained from all participants prior to the study.

### 2.3. Clinical Samples

Fresh tumor tissues and adjacent normal liver tissues were collected from 60 HCC patients who underwent surgical resection at the First Hospital of Qinhuangdao between March 2019 and February 2021. The study was approved by the Institutional Review Board (2019‐033) and conducted in accordance with the Declaration of Helsinki. Written informed consent was obtained from all participants. Inclusion criteria were as follows: (1) histopathologically confirmed HCC diagnosis, (2) age 18–75 years, (3) no prior chemotherapy or radiotherapy, (4) adequate liver function (Child–Pugh Class A or B), and (5) Eastern Cooperative Oncology Group performance status 0–2. Exclusion criteria included the following: (1) recurrent HCC; (2) other concurrent malignancies; (3) severe cardiovascular, pulmonary, or renal dysfunction; (4) active infections; and (5) insufficient tissue samples for analysis. The cohort comprised 35 males and 25 females with a median age of 51 years (range: 35–70 years). All tissue samples were immediately snap‐frozen in liquid nitrogen and stored at −80°C until analysis. Adjacent normal tissues were obtained at least 2 cm away from the tumor margin and confirmed to be free of malignant cells by histopathological examination.

### 2.4. Quantitative Real‐Time PCR (qRT‐PCR)

Cell line RNA was isolated using an RNA extraction kit from Thermo Fisher and subsequently reverse‐transcribed into cDNA using Takara’s protocol according to the manufacturer’s instructions. Following the preparation of the cDNA library, qRT‐PCR was performed to assess P3H4 expression levels. The primers for the qRT‐PCR analysis were as follows: P3H4 forward: ACGCGCTGTTCAAGGCTAA, P3H4 reverse: CCAGCATCCCCTGATAGTAGT; GAPDH forward: GGAGCGAGATCCCTCCAAAAT, GAPDH reverse: GGCTGTTGTCATACTTCTCATGG.

### 2.5. Western Blotting

Cell lysates were collected using 1X RIPA Buffer supplemented with beta‐mercaptoethanol and PMSF, followed by the separation of total proteins via SDS‐PAGE. The proteins were subsequently transferred onto PVDF membranes. After blocking the membranes with nonfat milk for 40 min, they were incubated with primary antibodies at 4°C overnight. Following this incubation, the membranes were treated with HRP‐conjugated secondary antibodies, and the signals were detected using the Odyssey scanning system. GAPDH was utilized as the loading control. The antibodies employed in this study included P3H4 (Rabbit, Cell Signaling Technology, Massachusetts, United States, 1:1000), Hexokinase 2 (HK2) (Rabbit, Cell Signaling Technology, Massachusetts, United States, 1:1000), Lactate Dehydrogenase A (LDHA) (Rabbit, Cell Signaling Technology, Massachusetts, United States, 1:1000), PI3K (Mouse, Santa Cruz, California, United States, 1:200), p‐PI3K (Rabbit, Cell Signaling Technology, Massachusetts, United States, 1:1000), AKT (Mouse, Santa Cruz, California, United States, 1:200), p‐AKT (Rabbit, Cell Signaling Technology, Massachusetts, United States, 1:1000), and GAPDH (Mouse, Santa Cruz, California, United States, 1:1000).

### 2.6. Lentivirus‐Mediated Short Hairpin RNA (shRNA) Knockdown (KD)

shRNA oligonucleotides were obtained from MilliporeSigma, with the target sequences as follows: sh‐NC CCTAAGGTTAAGTCGCCCTCG, sh‐P3H4#1 CACATGTACCTGCAGTCAGAT, sh‐P3H4#2 GCAGCAGCAAGAACTATTTAT, and sh‐P3H4#3 GCAGTACGAGAAGTACAGCTT. The sense and antisense sequences were combined into pairs of complementary oligonucleotides, which were subsequently annealed and cloned into pLKO.1 plasmids obtained from Addgene. The plasmids were transformed into bacterial cells, subjected to midi‐preparation, and verified through sequencing. Finally, they were packaged with pVSV‐G and psPAX2 packaging vectors (Addgene) for lentivirus production in 239 T cells. The collected virus was used to infect HCC cells with an MOI of 10 in the presence of 8 *μ*g/mL of polybrene for 48 h. The infected cells with stable shRNA expression were selected with 1 *μ*g/mL puromycin for 10 days.

### 2.7. Cell Growth Assay

Cell growth was assessed using the CCK8 kit (Solarbio Science & Technology, China) according to the manufacturer’s instructions. Cells were seeded in 96‐well plates at a density of 10,000 cells per well and cultured in complete medium. Absorbance measurements were taken at 450 nm using a spectrophotometer at 0, 24, 48, and 72 h postseeding.

### 2.8. 5‐Ethynyl‐2 ^′^‐Deoxyuridine (EdU) Cell Proliferation Assay

Cells were seeded on coverslips and cultured in complete medium until reaching 60%–70% confluency. Proliferating cells were labeled by incubating with 10 *μ*M EdU for 4 h. Following incubation, cells were fixed with 4% paraformaldehyde and permeabilized with 0.3% Triton X‐100. The EdU detection was performed by incubating the coverslips with the click reaction buffer (containing azide dye, copper sulfate, and ascorbic acid) for 30 min at room temperature in the dark. Nuclei were counterstained with DAPI (4 ^′^,6‐diamidino‐2‐phenylindole) for 10 min at room temperature, protected from light. After washing three times with PBS, coverslips were mounted on glass slides using an antifade mounting medium. Images were acquired using a fluorescence microscope. Cell proliferation rate was calculated by determining the percentage of EdU‐positive cells among the total DAPI‐positive cells.

### 2.9. Glycolytic Activity Assessment

Glycolytic parameters were measured using commercial assay kits from Abcam (Cambridge, United Kingdom) according to the manufacturer’s instructions. The analysis included glucose uptake assay (ab136955), L‐lactate production assay (ab65331), and ATP detection assay (ab113849). These measurements were performed in both Huh7 and Hep3B cell lines, comparing the glycolytic activity between cells expressing control shRNA (sh‐NC) and P3H4‐KD cells (sh‐P3H4). Briefly, cells were cultured under standard conditions, harvested at designated time points, and processed according to each assay’s specific protocol. Results were normalized to cell number or protein content and presented as relative values compared to the control group.

### 2.10. Nude Mouse Tumorigenicity Assay

This study was approved by the Animal Use and Care Committee of First Hospital of Qinhuangdao (DL2024‐078) and conducted in compliance with the ARRIVE guidelines (https://arriveguidelines.org). Twelve 6‐week‐old male BALB/c nude mice were obtained from SLAC Experimental Animal Laboratory (Shanghai, China) and randomly divided into two groups (*n* = 6 per group): sh‐NC (control) and sh‐P3H4 (KD) groups. Each mouse was subcutaneously injected with 100 *μ*L cell suspension containing 5 × 10^6^ Huh7 cells (either sh‐NC or sh‐P3H4 transfected) on the posterior flank. Tumor growth was monitored regularly, and once tumors became palpable (approximately Day 7 postinjection), tumor dimensions were measured every 4 days using digital calipers. Tumor volume was calculated using the formula: *V* = (length × width^2^)/2. On Day 28 postinjection, mice were humanely euthanized by CO_2_ asphyxiation followed by cervical dislocation as a secondary method to ensure death. CO_2_ was administered at a flow rate of 10%–30% of the chamber volume per minute until cessation of respiratory movement, followed by an additional 1–2 min of CO_2_ exposure. Cervical dislocation was then performed to confirm euthanasia before tumor excision. Tumor tissues were collected and subsequently fixed in 10% neutral buffered formalin, embedded in paraffin, and sectioned for immunohistochemistry (IHC) analysis to evaluate the expression of relevant proteins.

### 2.11. IHC Analysis

Formalin‐fixed, paraffin‐embedded tumor samples were sectioned at 4 *μ*m thickness for immunohistochemical analysis of Ki67 expression. Sections were deparaffinized in xylene and rehydrated through a graded alcohol series. Antigen retrieval was performed using an autoclave method (121°C for 10 min in 10 mM citrate buffer, pH 6.0). Endogenous peroxidase activity was quenched by incubating sections with 3% hydrogen peroxide for 10 min. After blocking with 5% normal goat serum, sections were incubated with primary anti‐Ki67 antibody (1:200 dilution, Abcam, ab15580) overnight at 4°C. Detection was performed using a streptavidin–peroxidase conjugate secondary antibody system and visualized with 3,3 ^′^‐diaminobenzidine (DAB) chromogen, followed by hematoxylin counterstaining. Ki67 expression was quantified by examining at least five high‐power fields (400×) in regions of highest staining intensity. The proliferation index was calculated as the percentage of tumor cells showing positive nuclear staining for Ki67 relative to the total number of tumor cells counted per field. All assessments were performed by two independent observers blinded to the experimental groups.

### 2.12. Statistical Analysis

Statistical analyses were conducted using GraphPad Prism Version 7.0 software (GraphPad Software, California, United States). Error bars represent the mean ± SEM. The differences between the two groups were assessed using a two‐tailed Student’s *t*‐test, while comparisons among multiple groups were performed using a one‐way ANOVA test. A *p* value of less than 0.05 was considered statistically significant.

## 3. Results

### 3.1. P3H4 Expression in HCC: Multidatabase Analysis and Clinical Validation

To investigate P3H4 expression in HCC, we conducted comprehensive analyses using multiple databases and clinical samples. Initial screening of the TCGA database, comprising 369 liver hepatocellular carcinoma (LIHC) patients and 160 normal liver samples through GEPIA, revealed significantly elevated P3H4 expression in HCC tumors compared to normal tissues (Figure 1a). For validation across databases, we examined the CPTAC LIHC database using UALCAN tools. This analysis confirmed our initial findings, demonstrating significantly higher P3H4 protein levels in liver tumor tissues compared to normal tissues (Figure 1b). The consistency across independent databases strengthens the reliability of these observations. Survival analysis using the Kaplan–Meier plotter revealed a significant correlation between elevated P3H4 expression and poor prognosis, suggesting that P3H4 may serve as a negative prognostic indicator in HCC patients (Figure 1c).

Figure 1P3H4 is overexpressed in hepatocellular carcinoma and correlates with poor prognosis. (a) Significantly elevated P3H4 mRNA expression in LIHC patients compared to normal controls from the TCGA database. (b) Validation of increased P3H4 expression in HCC tissues using the CPTAC database. (c) Kaplan–Meier survival analysis showing significantly reduced overall survival in patients with high P3H4 expression. (d) qRT‐PCR confirmation of upregulated P3H4 mRNA expression in 60 paired HCC tissues compared to adjacent normal tissues. (e) Western blot analysis demonstrating enhanced P3H4 protein expression in three representative HCC tumor samples versus matched normal tissues. (f) Representative immunohistochemical staining revealing robust P3H4 expression in HCC tissues compared to adjacent normal tissues. (g) Clinical validation of poor prognosis in HCC patients with high P3H4 expression from our patient cohort.  ^∗^ *p* < 0.05 and  ^∗∗∗^ *p* < 0.001.

**Figure:**
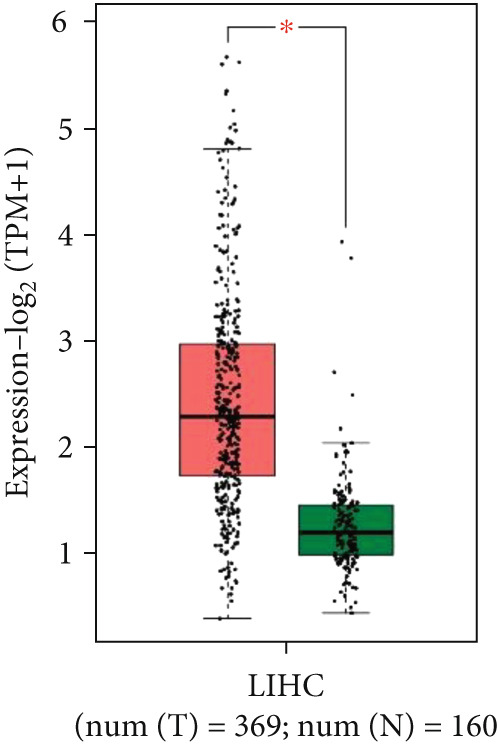
(a)

**Figure:**
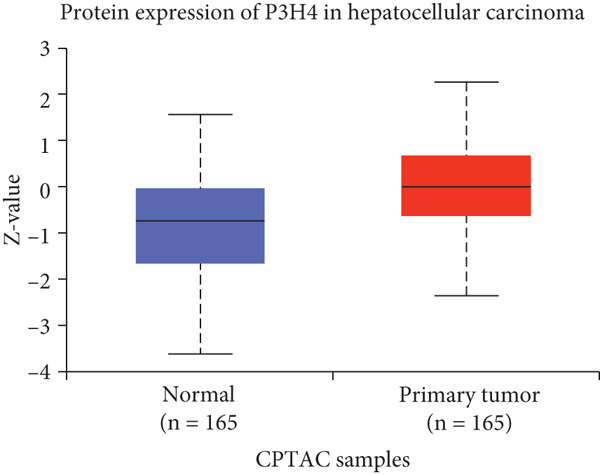
(b)

**Figure:**
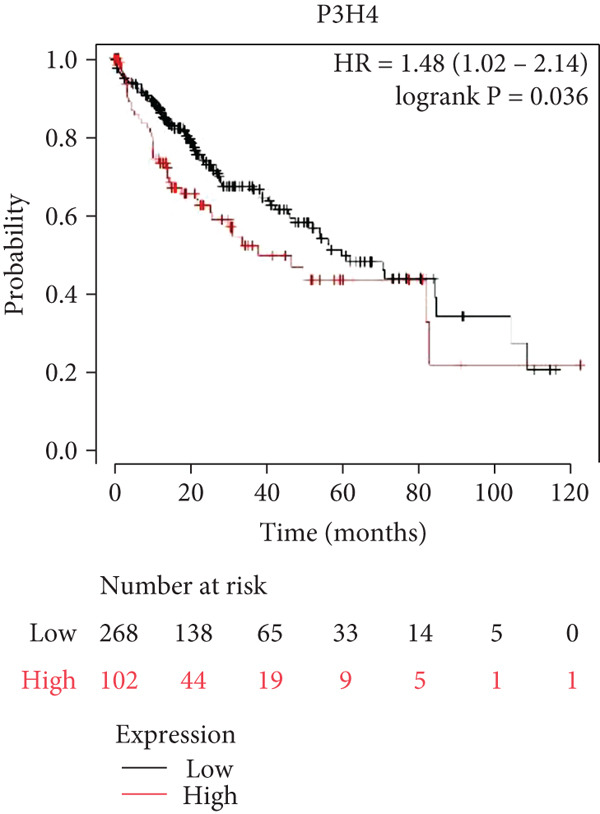
(c)

**Figure:**
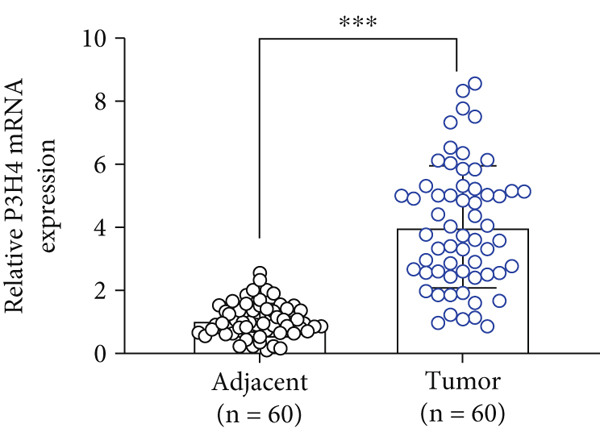
(d)

**Figure:**
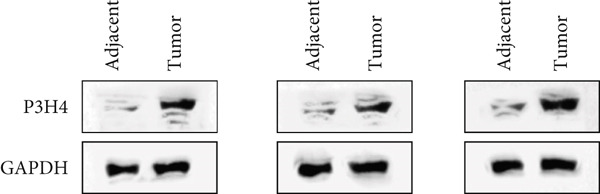
(e)

**Figure:**
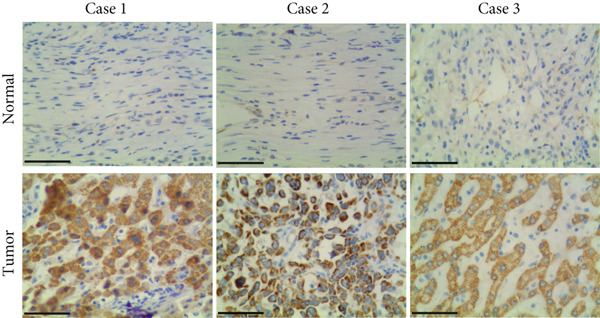
(f)

**Figure:**
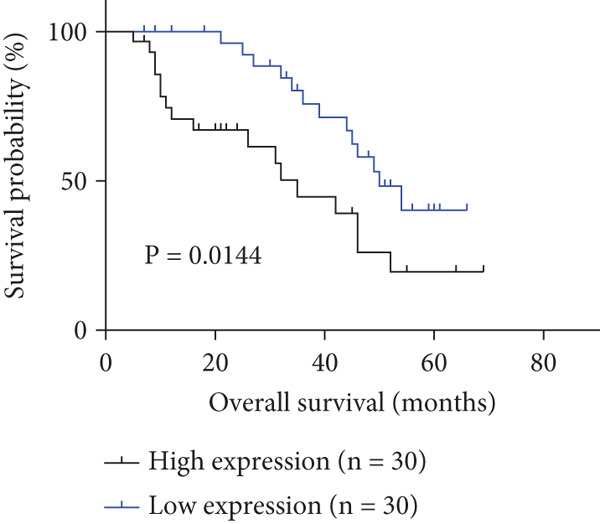
(g)

To clinically validate these bioinformatic findings, we analyzed samples from 60 HCC patients (Table 1). qRT‐PCR measurements demonstrated significantly higher P3H4 mRNA levels in tumor tissues compared to adjacent normal tissues, corroborating our database analyses (Figure 1d). Western blot analysis of three representative patient sample pairs further confirmed increased P3H4 protein expression in tumor tissues (Figure 1e). The elevated protein expression of P3H4 in tumor samples was further confirmed by IHC staining (Figure 1f). Finally, survival analysis of our clinical cohort, using median P3H4 expression as the cutoff, revealed that patients with higher P3H4 levels experienced significantly shorter OS times (Figure 1g). These results collectively suggest that P3H4 upregulation may contribute to HCC progression and could serve as a potential prognostic biomarker.

**Table 1:** Correlation between P3H4 expression and clinical characteristics of hepatocellular carcinoma patients.

**Clinical parameters**	**Patients**	**P3H4 expression**		**X** ^2^	**p** **value**
		**Low**	**High**		
Age (years)				0.30	0.58
<55	40	19	21		
≥55	20	11	9		
Gender				3.36	0.07
Male	35	21	14		
Female	25	9	16		
Tumor size				0.42	0.52
<5 cm	12	5	7		
≥5 cm	48	25	23		
Tumor number				1.83	0.18
Single	39	22	17		
Multiple	21	8	13		
Edmondson grade				5.08	0.02
I + II	42	25	17		
III	18	5	13		
Metastasis				4.81	0.03
Negative	13	10	3		
Positive	47	20	27		
Microvascular invasion				8.08	0.004
Absence	31	21	10		
Presence	29	9	20		
AFP (*μ*g/L)				6.67	0.01
<50	30	20	10		
≥50	30	10	20		
Cirrhosis				0.74	0.39
Negative	17	7	10		
Positive	43	23	20		

### 3.2. Correlations Between P3H4 Expression Levels and Clinicopathological Characteristics in HCC

We next investigated the correlation between P3H4 expression and clinicopathological characteristics in HCC patients. To this end, we analyzed parameters from 60 patients using the chi‐square method [18]. Initially, we established the cutoff value based on the median P3H4 expression in the HCC group and divided the patients into two groups: the P3H4 low expression group (*n* = 30) and the high expression group (*n* = 30). Subsequently, we performed a chi‐square test to assess the correlations between P3H4 expression and various clinical parameters. Our analysis revealed that P3H4 expression levels were positively and significantly associated with the Edmondson grade, metastasis, microvascular invasion, and alpha‐fetoprotein (AFP) levels. However, no significant correlations were found between P3H4 expression and patients’ age, gender, tumor size, tumor number, and cirrhosis (Table 1).

### 3.3. Knocking Down P3H4 Suppresses Proliferation and Invasion of HCC Cells

Next, we compared the normal hepatocyte line THLE‐3 with HCC cell lines, including Huh7, HCCLM3, and Hep3B, via qRT‐PCR to investigate the expression levels of P3H4 in different cell lines. The results indicated that P3H4 was expressed at significantly higher levels in all HCC cell lines compared to the normal liver cell line (Figure 2a). To further explore the function of P3H4 in HCC, we selected the two cell lines with the highest P3H4 expression, Huh7 and Hep3B, as our experimental models. We designed three shRNAs to knock down P3H4 and established stable HCC P3H4‐KD cell lines. All three shRNAs demonstrated KD effects compared to the negative control (sh‐NC) in both HCC cell lines, with sh‐P3H4#1 showing the highest efficiency (Figure 2b). Thus, we selected sh‐P3H4#1 for subsequent functional assays. To assess the impact of P3H4 on HCC cell proliferation, we conducted CCK8 assays in Huh7 and Hep3B cell lines, measuring absorbance at four time points (0, 24, 48, and 72 h) at a wavelength of 450 nm for both the sh‐NC and sh‐P3H4 groups. The CCK8 results, presented in Figure 2c, demonstrated that knocking down P3H4 significantly inhibited cell growth in both cell lines. EDU labeling assay for DNA synthesis detection further confirmed that P3H4 KD drastically reduced the percentage of cells actively synthesizing DNA (Figure 2d). Furthermore, P3H4 KD significantly suppressed the invasive capacities of Huh7 and Hep3B cells in transwell chambers coated with Matrigel (Figure 2e). In conclusion, these results indicate that P3H4 expression is required to sustain the proliferation and invasion of HCC cell lines in vitro.

Figure 2P3H4 knockdown inhibits the proliferation and invasion of HCC cells in vitro. (a) Comparative analysis of P3H4 expression across normal hepatocytes (THLE‐3) and HCC cell lines (Huh7, HCCLM3, and Hep3B) by qRT‐PCR. (b) Efficient knockdown of P3H4 expression using three different shRNAs in Huh7 and Hep3B cells, with sh‐P3H4#1 showing the highest efficiency. (c) CCK8 assay demonstrating significantly reduced proliferation in P3H4‐silenced Huh7 and Hep3B cells compared to control cells. (d) DNA synthesis evaluation by EdU labeling assay showing a marked decrease in proliferating cells following P3H4 knockdown. (e) Invasive capacity assessment by Transwell assay revealing substantially impaired invasion in P3H4‐silenced HCC cells.  ^∗^ *p* < 0.05,  ^∗∗^ *p* < 0.01, and  ^∗∗∗^ *p* < 0.001.

**Figure:**
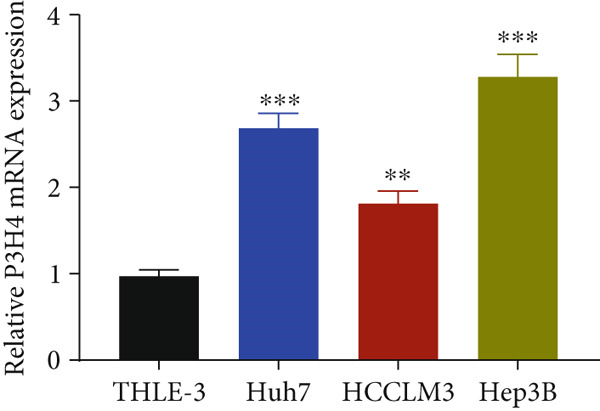
(a)

**Figure:**
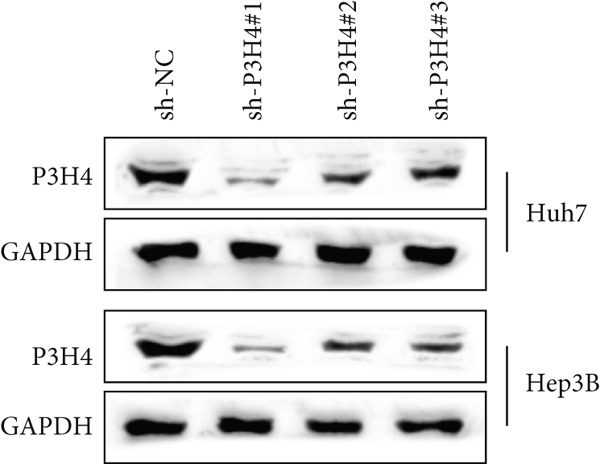
(b)

**Figure:**
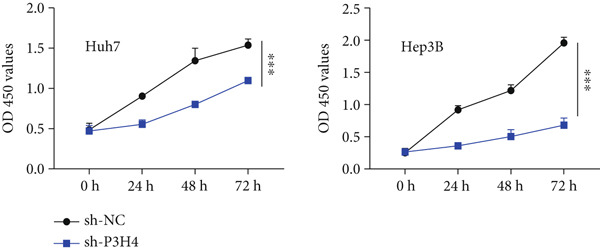
(c)

**Figure:**
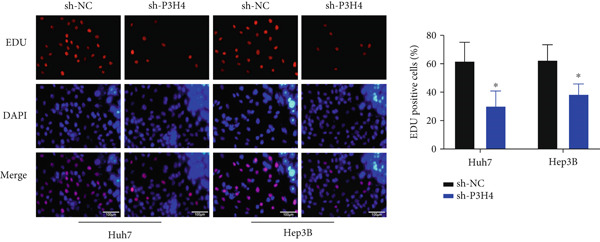
(d)

**Figure:**
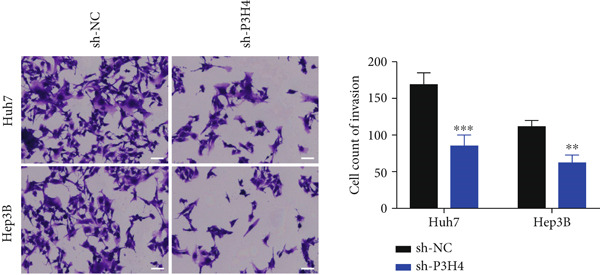
(e)

### 3.4. Knocking Down P3H4 Suppresses Glycolysis in HCC Cells

Given that HCC cells primarily utilize glucose for energy during proliferation and invasion [17], we investigated whether P3H4 plays a role in glycolytic metabolism in HCC cells. We conducted comprehensive glycolytic function assays in two HCC cell lines (Huh7 and Hep3B) following P3H4 KD (sh‐P3H4) compared to control (sh‐NC) conditions. P3H4 KD significantly reduced glucose consumption in both Huh7 and Hep3B cells compared to their respective control groups (Figure 3a). Concordantly, lactate production, an end product of aerobic glycolysis characteristic of cancer cells, was markedly decreased in sh‐P3H4 cells (Figure 3b). ATP levels, representing cellular energy production, were also significantly diminished in P3H4‐silenced cells (Figure 3c), suggesting compromised bioenergetic capacity. Western blot analysis revealed that P3H4 KD substantially reduced the expression of HK2, which catalyzes the first rate‐limiting step of glucose metabolism, and LDHA, which converts pyruvate to lactate, in both Huh7 and Hep3B cell lines (Figure 3d). These findings collectively demonstrate that P3H4 positively regulates glycolytic metabolism in HCC cells, suggesting a potential mechanism by which P3H4 promotes HCC progression through metabolic reprogramming.

Figure 3P3H4 silencing disrupts glycolytic metabolism in HCC cells. Metabolic analysis showing significantly decreased (a) glucose consumption, (b) lactate production, and (c) ATP levels in P3H4‐knockdown Huh7 and Hep3B cells compared to control cells. (d) Western blot analysis revealing downregulation of key glycolytic enzymes HK2 and LDHA following P3H4 silencing in both HCC cell lines.  ^∗∗^ *p* < 0.01 and  ^∗∗∗^ *p* < 0.001.

**Figure:**
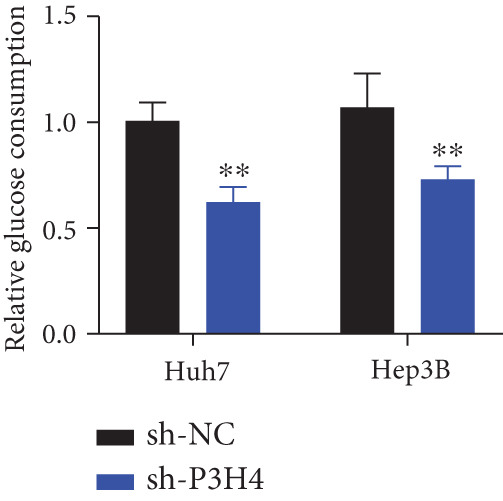
(a)

**Figure:**
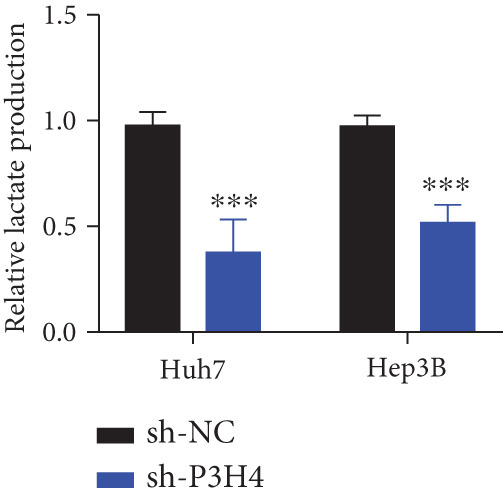
(b)

**Figure:**
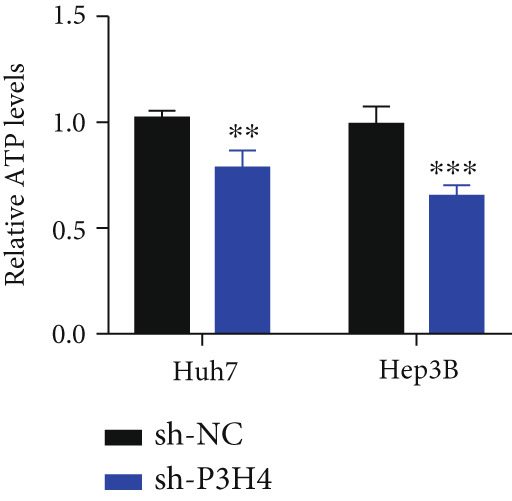
(c)

**Figure:**
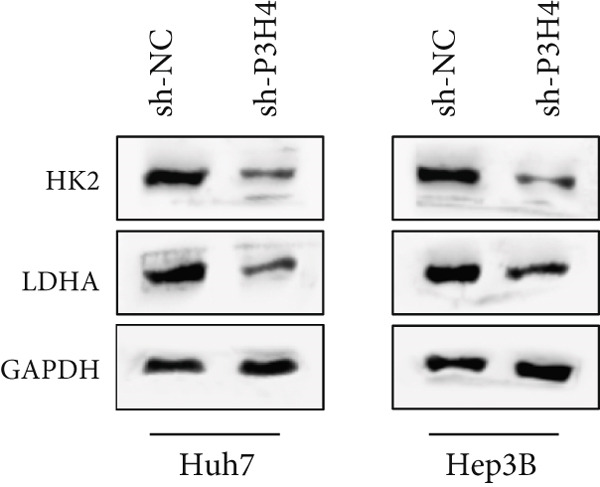
(d)

### 3.5. Knocking Down P3H4 Inhibits the PI3K/AKT Pathway

In HCC, the PI3K/AKT pathway has been reported to promote glycolytic metabolic reprogramming in cancer progression [17]. In our study, we also found that knocking down P3H4 led to the dephosphorylation of both PI3K and AKT (Figure 4). These results were confirmed in both Huh7 and Hep3B cell lines, suggesting that P3H4 plays a role in maintaining the activity of the PI3K/AKT pathway during HCC progression.

Figure 4P3H4 knockdown attenuates PI3K/AKT signaling pathway in HCC cells. Western blot analysis demonstrating markedly reduced phosphorylation levels of PI3K and AKT in P3H4‐silenced (a) Huh7 and (b) Hep3B cells, while total protein levels remain unchanged.

**Figure:**
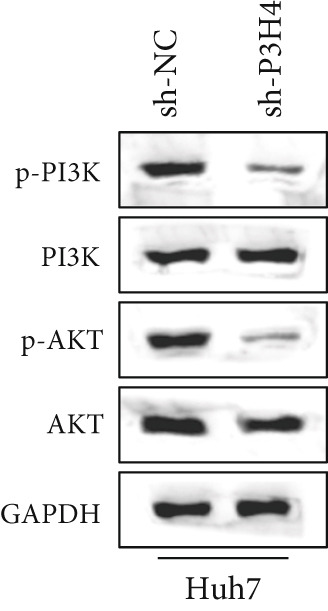
(a)

**Figure:**
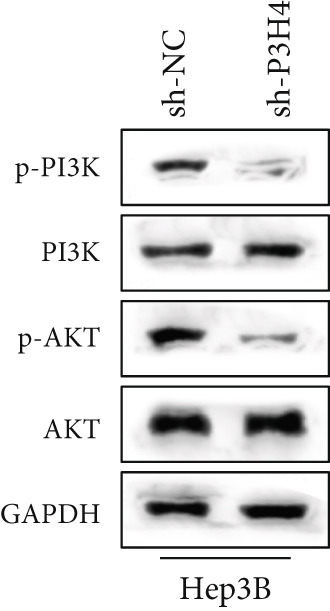
(b)

### 3.6. P3H4 KD Impairs HCC Tumor Growth In Vivo

To validate P3H4’s role in HCC progression in vivo, we established a xenograft model using Huh7 cells with P3H4 KD (sh‐P3H4) or control (sh‐NC) injected subcutaneously into nude mice. Tumor development was significantly impaired in the P3H4‐silenced group, with markedly reduced tumor volumes at 28 days postinjection compared to controls (Figure 5a). Similarly, final tumor weights were significantly lower in the sh‐P3H4 group (Figure 5b). Immunohistochemical analysis revealed decreased Ki‐67 expression in P3H4‐KD tumors, indicating reduced cell proliferation (Figure 5c). Furthermore, we detected significantly reduced levels of glycolytic enzymes (HK2 and LDHA) and markedly attenuated phosphorylation of PI3K and AKT in tumor samples with P3H4 silencing (Figure 5d). Collectively, these findings confirm that P3H4 silencing effectively suppresses HCC tumor growth in vivo, highlighting the critical role of P3H4 in maintaining PI3K/AKT pathway activation and sustaining glycolytic metabolism during HCC progression.

Figure 5P3H4 silencing suppresses HCC tumor growth and glycolytic signaling in vivo. (a) Tumor growth curves showing significantly reduced tumor volumes in mice bearing P3H4‐knockdown Huh7 xenografts compared to the control group. (b) Final tumor weight measurement confirming impaired tumor growth in the sh‐P3H4 group. (c) Immunohistochemical analysis of Ki67 expression revealing decreased proliferation index in P3H4‐silenced tumors. (d) Western blot analysis of xenograft tissues showing concurrent downregulation of glycolytic enzymes (HK2 and LDHA) and diminished phosphorylation of PI3K/AKT pathway components in P3H4‐knockdown tumors.  ^∗^ *p* < 0.05 and  ^∗∗∗^ *p* < 0.001.

**Figure:**
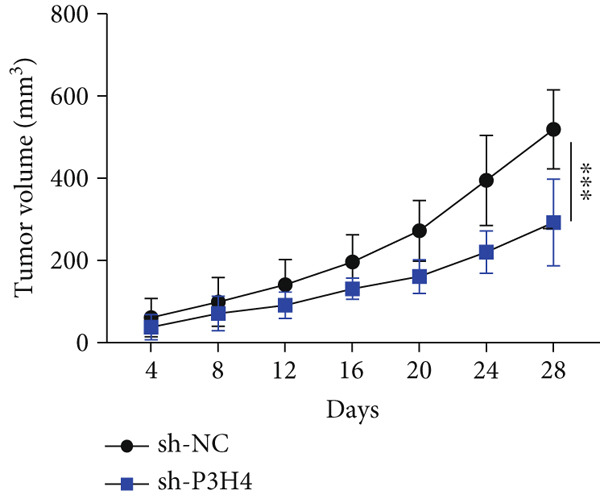
(a)

**Figure:**
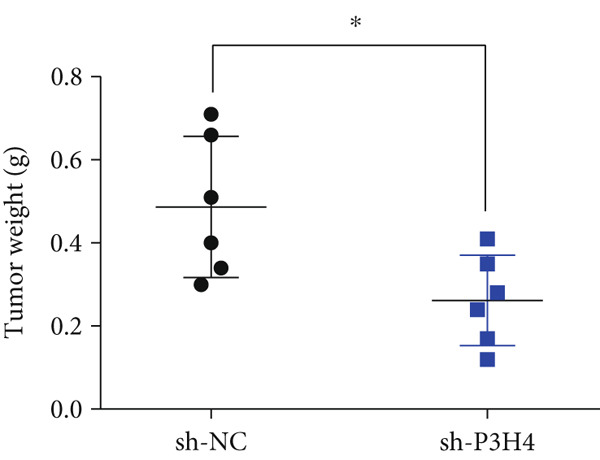
(b)

**Figure:**
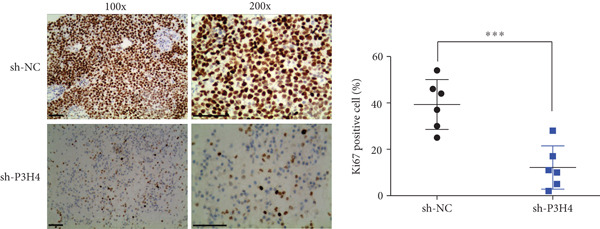
(c)

**Figure:**
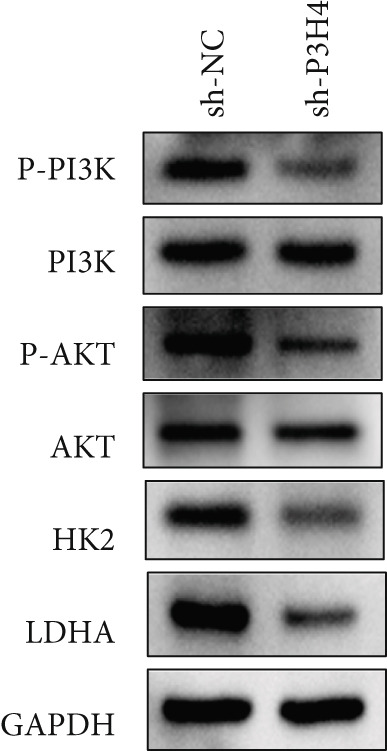
(d)

## 4. Discussion

HCC continues to challenge global health systems with devastating consequences for human health, emphasizing the urgent need to elucidate its molecular mechanisms. Building on previous research showing P3H4 overexpression in bladder, lung, and kidney cancers [13–15], our study provides the first evidence of P3H4’s crucial role in HCC pathogenesis. We demonstrate that P3H4 is significantly upregulated in HCC tissues compared to normal liver tissues, with elevated expression correlating with poorer patient survival. Our functional investigations reveal that P3H4 silencing inhibits HCC cell proliferation and invasion in vitro; disrupts glycolytic metabolism by reducing glucose consumption, lactate production, and ATP generation; and downregulates key glycolytic enzymes HK2 and LDHA. These findings are validated in vivo, where P3H4 KD significantly suppresses tumor growth in xenograft models, collectively establishing P3H4 as a potential therapeutic target for HCC treatment.

The Warburg effect, characterized by increased aerobic glycolysis, is a hallmark of metabolic reprogramming in HCC that provides cancer cells with energy and biosynthetic intermediates necessary for rapid proliferation [19, 20]. Our findings reveal P3H4 as a novel regulator of glycolytic metabolism in HCC, where its KD significantly reduced glucose consumption, lactate production, and ATP levels while decreasing expression of key glycolytic enzymes HK2 and LDHA. These results align with previous studies showing that targeting glycolytic enzymes can effectively suppress HCC progression [21, 22]. The metabolic control exerted by P3H4 provides a mechanistic explanation for its effects on cell proliferation and tumor growth, suggesting that P3H4 may promote HCC development partly through enhancing glycolytic flux to meet the increased energy demands of cancer cells.

The PI3K/AKT pathway plays a pivotal role in regulating cellular metabolism and is frequently dysregulated in HCC, contributing to enhanced glycolysis and tumor progression [23, 24]. This signaling cascade activates downstream effectors that directly upregulate glucose transporters and glycolytic enzymes, thereby promoting the Warburg effect [25]. Our study demonstrates that P3H4 KD coincides with reduced expression of glycolytic enzymes HK2 and LDHA, suggesting potential crosstalk between P3H4 and the PI3K/AKT pathway in HCC. Previous investigations have shown that inhibition of PI3K/AKT signaling can reverse the glycolytic phenotype and suppress HCC growth [26–28]. Future studies should explore whether P3H4 exerts its proglycolytic effects through modulation of PI3K/AKT activity, which could provide additional therapeutic opportunities by targeting this metabolic vulnerability in HCC.

Despite the promising findings, our study has several limitations. First, the molecular mechanisms by which P3H4 regulates glycolytic enzymes remain to be fully elucidated, particularly regarding direct or indirect interactions with the PI3K/AKT pathway. Second, our investigation focused primarily on in vitro cell models and subcutaneous xenografts, which may not fully recapitulate the complex tumor microenvironment of human HCC. Third, rescue experiments involving P3H4 overexpression combined with glycolysis inhibitors or PI3K/AKT pathway blockers would provide more definitive evidence of the functional relationship between P3H4 and metabolic reprogramming in HCC. Furthermore, the potential correlation between P3H4 expression and clinical parameters beyond survival, such as tumor stage, grade, and treatment response, warrants further investigation in larger patient cohorts. These data are valuable to inform whether P3H4 could serve as a reliable biomarker for HCC diagnosis or monitoring treatment response. In addition, the role of P3H4 in other metabolic pathways beyond glycolysis, including glutaminolysis and lipid metabolism, was not explored in this study. The development of specific P3H4 inhibitors would be necessary to evaluate their therapeutic potential in preclinical and clinical settings.

## 5. Conclusion

In conclusion, our study suggests that P3H4 functions as an oncogenic driver in HCC by promoting cell proliferation, invasion, and glycolytic reprogramming. The significant correlation between elevated P3H4 expression and poor patient prognosis underscores its clinical relevance. P3H4 silencing suppresses tumor growth both in vitro and in vivo while simultaneously downregulating key glycolytic enzymes HK2 and LDHA, suggesting a potential metabolic vulnerability that could be therapeutically exploited. Although the precise mechanisms through which P3H4 orchestrates these malignant processes require further investigation, particularly regarding its potential interplay with the PI3K/AKT pathway, our findings establish P3H4 as a promising therapeutic target and prognostic biomarker for HCC patients.

**Nomenclature**
CCK8: Cell Counting Kit 8
CPTAC: The Clinical Proteomic Tumor Analysis Consortium
HCC: hepatocellular carcinoma
TCGA: The Cancer Genome Atlas

## Ethics Statement

The study was approved by the Ethics Committee of First Hospital of Qinhuangdao. All methods were performed in accordance with the Declaration of Helsinki. The animal experiment was approved by the Animal Care and carried out in compliance with the ARRIVE guidelines (https://arriveguidelines.org).

## Consent

Written informed consents were signed by all participants before the study.

## Disclosure

All authors read and approved the final manuscript.

## Conflicts of Interest

The authors declare no conflicts of interest.

## Author Contributions

Lili Yan and Ji Lv mainly participated in literature search, study design, writing, and critical revision and prepared Figures 1, 2, 3, 4, and 5. Meimei Xu, Hongyu Jia, Shanshan Li, and Zhihui Tan mainly participated in data collection, data analysis, and data interpretation and prepared Table 1. Yandan Fan performed the revision experiments, analyzed the data, and organized the figures. Chunyan Lin supervised the revision, revised the manuscript, and drafted the response letter. Lili Yan and Yandan Fan Fan contributed equally to this work.

## Funding

This study was supported by the Fujian Provincial Natural Science Foundation General Program (No. 2021J011388 and 2021J011390).

## Data Availability

The datasets generated and/or analyzed during the current study are available from the corresponding author on reasonable request.
